# Targeting Chemokine Receptor CCR4 in Adult T-Cell Leukemia-Lymphoma and Other T-Cell Lymphomas

**DOI:** 10.1007/s11899-012-0124-3

**Published:** 2012-04-27

**Authors:** Kensei Tobinai, Takeshi Takahashi, Shiro Akinaga

**Affiliations:** 1 Department of Hematology, and Hematopoietic Stem Cell Transplantation, National Cancer Center Hospital, 5-1-1 Tsukiji, Chuo-ku, Tokyo, 104-0045 Japan; 2 Clinical Development Department, Kyowa Hakko Kirin Co., Ltd, 1-6-1 Ohtemachi, Chiyoda-ku, Tokyo, 100-8185 Japan; 3 Development Division, Kyowa Hakko Kirin Co., Ltd, 1-6-1 Ohtemachi, Chiyoda-ku, Tokyo, 100-8185 Japan

**Keywords:** Chemokine receptor, CCR4, Adult T-cell leukemia-lymphoma, ATL, Peripheral T-cell lymphoma, PTCL, Monoclonal antibody, Mogamulizumab, KW-0761

## Abstract

Peripheral T-cell lymphoma (PTCL) is a group of lymphoid malignancy that remains difficult to treat, as most PTCL becomes refractory or relapses, and thus there is an unmet medical need for novel treatment modalities. CC chemokine receptor 4 (CCR4) is expressed in various types of PTCL including adult T-cell leukemia-lymphoma (ATL), which has the worst prognosis among them. A phase II study of a defucosylated, humanized anti-CCR4 monoclonal antibody, mogamulizumab (KW-0761), yielded an overall response rate of 50 % (13/26) and a median progression-free survival of 5.2 months in relapsed patients with CCR4-positive ATL who had been previously treated with chemotherapy. Mogamulizumab also showed potential efficacy for cutaneous T-cell lymphoma in a US phase I/II study. Further preclinical and clinical investigations are needed to examine whether concomitant use of this novel agent with other agents with different mechanisms of action would be more effective for ATL and other PTCLs.

## Introduction

Peripheral T-cell lymphoma (PTCL) represents a small, heterogeneous group of non-Hodgkin lymphoma (NHL) which is derived from more mature T-cells and natural killer (NK) cells, and accounts for approximately 10 % of NHL cases in Western countries [1, 2] and for approximately 20 %–25 % of those in Japan [3, 4]. PTCL, a collective entity of nearly 20 different subtypes defined according to morphology, immunophenotype, genotype, and clinical features [5], can be largely classified into the following two groups according to clinical features including the sites of lesions: (1) cutaneous T-cell lymphoma (CTCL), which is the general term for diseases that initially or mainly occur in the skin, and (2) PTCL other than CTCL. Treatment strategies have been separately developed for these two groups [6].

Treatment options are substantially different for B-cell and T-cell lymphomas. Rituximab, an anti-CD20 monoclonal antibody, was developed for the treatment of B-cell lymphomas. The introduction of this agent into clinical practice has greatly improved the prognosis of patients with B-cell lymphoma [7]. Recently, bendamustine, which has little cross resistance with other chemotherapeutic agents presumably associated with its unique chemical structure of an alkylating agent and a nucleoside analog, has been developed as effective treatment of relapsed or refractory B-cell lymphoma, considering its lack of cross resistance with other chemotherapeutic agents [8]. However, PTCL remains extremely difficult to treat, because most PTCL subtypes become refractory to even aggressive chemotherapy regimens or relapse, with the exception of anaplastic lymphoma kinase-positive anaplastic large cell lymphoma (ALK^+^ ALCL), which responds well to the cyclophosphamide, doxorubicin, vincristine, and prednisone (CHOP) regimen [9]. Among the various entities of PTCLs, adult T-cell leukemia-lymphoma (ATL) harbors the worst prognosis [10]. Here, we will discuss novel agents that have been developed for the treatment of ATL and other PTCLs, mainly focusing on mogamulizumab/KW-0761, which is a humanized monoclonal antibody targeting CC chemokine receptor 4 (CCR4) that has been actively developed for clinical use in Japan and the United States.

## PTCL and Novel Agents

PTCL-not otherwise specified (PTCL-NOS) and angioimmunoblastic T-cell lymphoma (AITL), which are the most common subtypes of PTCL (PTCL-NOS, 26 %; AITL, 19 %), show a poor prognosis with 5-year overall survival (OS) and failure-free survival (FFS) of about 30 % and 20 %, respectively [10]. Several new agents have recently been developed for the treatment of PTCL, mainly in patients with relapsed or refractory disease. Such agents have various mechanisms of action, including an immunomodulator (lenalidomide), a proteasome inhibitor (bortezomib), histone deacetylase inhibitors (vorinostat, romidepsin, panobinostat), antifolate (pralatrexate), and biologics including antibodies and antibody-toxin/drug conjugates (alemtuzumab, siplizumab, denileukin diftitox, and brentuximab vedotin) as well as nucleoside analogs such as fludarabine, gemcitabine, nelarabine, and forodesine [11]. Of these agents, pralatrexate and romidepsin have been recently approved by the U.S. Food and Drug Administration (FDA) and are now being used in the U.S. for the treatment of relapsed or refractory PTCL. In 2011, brentuximab vedotin (formerly known as SGN-35) was also approved for the treatment of relapsed or refractory ALCL and Hodgkin lymphoma.

ATL has the worst prognosis among PTCL, with 5-year OS and FFS of 14 % and 12 %, respectively [10]. ATL is a peripheral T-cell malignancy associated with human T-cell lymphotropic virus type I (HTLV-1), and is relatively frequent in southwestern Japan, West Africa, the Caribbean islands, and Brazil, which are HTLV-1 endemic areas [12]. It is estimated that there are about 1.2 million HTLV-1 carriers in Japan, of whom a few percent develop ATL [13], and approximately 700 to 1000 people die of this disease per year [14]. ATL is classified into four disease subtypes (acute, lymphoma, chronic, and smoldering), based on clinical features including leukemic changes, high lactate dehydrogenase, hypercalcemia and organ infiltration, and the median survival time varies according to the disease type: acute type, 6 months; lymphoma type, 10 months; chronic type, 24 months; and smoldering type, 3 years or more [15]. It is recommended that treatment strategies should be selected according to the disease subtype [15]. In Japan, the acute type, lymphoma type, and chronic type with unfavorable prognostic factors have been regarded as aggressive ATL subtypes requiring immediate treatment, and intensive combination chemotherapy or allogeneic hematopoietic stem-cell transplantation are generally recommended therapeutic options [16].

The Japan Clinical Oncology Group-Lymphoma Study Group (JCOG-LSG) has been investigating the efficacy of combination chemotherapy for aggressive lymphomas including ATL or for ATL alone since the early 1980s. At the start of the investigation, CHOP-like regimens were evaluated because ATL was considered to be a type of NHL, but the outcome was poor [17]. Then, the LSG15 regimen consisting of the drugs used in the CHOP regimen plus four other drugs (ranimustine, vindesine, etoposide, and carboplatin) with the prophylactic use of granulocyte colony-stimulating factor (G-CSF) was evaluated. In a phase III trial, JCOG9801, this dose-intensified multiagent chemotherapy regimen was shown to be more effective than CHOP-14 regimen, with a complete response rate of 40 %, 3-year OS of 24 %, and median survival time of 12.7 months [18, 19]. However, since the outcome of this dose-intensified regimen was still inferior to that in other PTCLs and B-cell lymphomas, further improvement is necessary. In Western countries, combination therapy with interferon-α and zidovudine has been widely used for all disease subtypes of ATL. A recently published meta-analysis suggested the effectiveness of this combination therapy for ATL, especially leukemic forms such as acute and chronic types [20].

Several new antibodies are currently under development for the treatment of T-cell lymphoma. They are based on the unique immunophenotypic features of ATL cells, which express mature T-cell antigens such as CD2, CD25 (interleukin [IL]-2 receptor), and CD52. Because of the unique intense expression of CD25 compared to that in other PTCL, monoclonal antibodies targeting the IL-2 receptor (anti-Tac), either radiolabeled or unlabelled (daclizumab), have been tested in patients with relapsed or refractory ATL. However, the clinical efficacy appears to be limited [21]. An anti-CD2 monoclonal antibody (siplizumab) [22], anti-CD52 antibody (alemtuzumab) [23, 24], and anti-transferrin receptor antibody (A24) [25] are also under development, but data are currently limited.

## Currently Available Therapeutic Agents for ATL

Pentostatin and sobuzoxane are chemotherapeutic agents that were previously approved for the treatment of ATL in Japan. Pentostatin, a purine nucleoside analog that inhibits adenosine deaminase, has been reported to be effective for T-cell malignancies, including T-cell prolymphocytic leukemia, CTCL, and PTCL [26]. The clinical efficacy of pentostatin was evaluated in patients with ATL from the 1980s to 1990s, and a phase II study of pentostatin revealed a response rate of 32 % (10 of 31) in patients with relapsed or refractory ATL [27]. Other drugs that are often used in patients with relapsed or refractory ATL are some combination chemotherapy regimens, including EPOCH (etoposide, prednisolone, vincristine, cyclophosphamide, and doxorubicin) and ESHAP (etoposide, methylprednisolone, high-dose cytarabine and cisplatin); however, there is no apparent evidence of an advantage of these combination chemotherapies over other therapeutic options. In the U.S., pralatrexate and romidepsin have been approved for the treatment of PTCL and can also be used for ATL. The efficacy of these drugs for ATL is not clear because they have been evaluated only in a very limited number of patients (the efficacy of pralatrexate was evaluated in a clinical study in one patient) [28].

## CCR4 as a Novel Therapeutic Target

Chemokines act as signaling molecules in the migration and tissue homing of various leukocytes. Among them, thymus and activation-regulated chemokine (TARC) and monocyte-derived chemokine (MDC) induce the selective recruitment of distinct subsets of T-cells through triggering of a chemokine receptor, CCR4. CCR4 is a seven-transmembrane G-protein coupled receptor and selectively expressed on Th2 cells and regulatory T cells [29, 30]. The expression on normal cells such as Th2 cells can be partly regulated by the ligand, especially MDC [31], while the regulation by the ligands on cancer cells are not yet understood. Ishida et al. analyzed 103 patients with ATL, and found that tumor cells from about 90 % of patients showed CCR4 expression [32]. They also found that patients with CCR4-positive ATL were more likely to have skin infiltration and had a worse outcome than those with CCR4-negative ATL, indicating that CCR4 played an important pathogenetic role in ATL [32]. In addition, Yoshie et al. found that the expression of CCR4 was increased in association of HTLV-1 and showed a relationship to Fra-2/Jun D which induces downstream genes such as c-Myb and SOX4, and MDM2 which promotes growth and inhibits apoptosis [33]. CCR4 is also expressed on other types of PTCL (29 % of total cases; PTCL-NOS, 38 %; AITL, 35 %; ALK^-^ ALCL, 67 %; mycosis fungoides [MF], 41 %) [34]. Jones et al. independently reported that some types of PTCL expressed CCR4, as well [35]. In addition, analysis of 50 patients with PTCL-NOS revealed that CCR4-positive patients had significantly shorter survival than CCR4-negative patients [34]. Nakagawa et al. analyzed 51 patients with PTCL-NOS using the array comparative genomic hybridization technique, and found that patients with PTCL-NOS with genomic aberrations had a significantly higher frequency of CCR4 positivity and a worse outcome than those with PTCL-NOS without genomic aberrations [36]. These findings resemble those observed in patients with ATL. Although the role of CCR4 in the tumorigenesis and progression of PTCL-NOS has not been fully elucidated, CCR4 seems to be a promising target molecule in the treatment of PTCL as well as in ATL.

## Clinical Trials of Mogamulizumab

Mogamulizumab/KW-0761 is a humanized monoclonal antibody that recognizes the N-terminal region of human CCR4 [37–39]. It is a therapeutic antibody produced using a novel glycoengineering technology that enhances antibody-dependent cellular cytotoxic (ADCC) activity [40]. Mogamulizumab and its human-mouse chimeric version, KM2760, showed potent antitumor activity mediated by enhanced ADCC against ATL cell lines and primary ATL cells in vitro and in vivo [39, 41, 42].

A phase I clinical study (0761–0501 Study: ClinicalTrials.gov Identifier NCT00355472) has been conducted in patients with CCR4-positive relapsed PTCL, including ATL [43•]. The primary objectives of the study were to assess the safety of mogamulizumab, and analyze its maximum tolerated dose (MTD) and pharmacokinetics. The secondary objectives were to determine the best overall response rate (ORR) and progression-free survival (PFS). Mogamulizumab was intravenously administered once a week for 4 weeks at four dose levels (0.01, 0.1, 0.5, and 1.0 mg/kg) according to the conventional 3 + 3 design. Enrolled in the study were 16 patients, of whom 13 had ATL (11 acute type, 2 lymphoma type), 1 had tumor-stage MF, and 2 had PTCL-NOS. All 16 patients receiving mogamulizumab were included in the safety and efficacy analyses. No dose-limiting toxicity (DLT) was observed in any of the 13 patients who received mogamulizumab at a dose of 0.01–1.0 mg/kg, and thus MTD was not reached. Then, three additional patients were enrolled to receive 1.0 mg/kg, the highest dose. One patient showed grade 4 neutropenia, grade 3 febrile neutropenia, and grade 3 skin eruption, but these adverse events occurred in only 1 of the 6 patients who received a dose of 1.0 mg/kg, indicating that this drug would be tolerated at least up to 1.0 mg/kg. The best ORR in the total 16 patients was 31 % (of those, 2 had complete response [CR] and 3 had partial response [PR]), and the best ORR was also 31 % in patients with ATL (of those, 2 had CR and another 2 had PR). Pharmacokinetic analysis revealed a plasma mogamulizumab trough concentration of 7.5–19.6 μg/mL after the 1st to 4th administration of mogamulizumab at a dose of 1.0 mg/kg. These concentrations were sufficient to kill primary ATL cells by ADCC activity in vitro (10 μg/mL). After the 4th administration of mogamulizumab at a dose of 1.0 mg/kg, its plasma half-life was approximately 18 days, which is comparable to the half-life (14 to 21 days) of endogenous human IgG. Lastly, although MTD was not reached, a tendency toward an increased incidence of grade 3 or higher toxicity was observed at 1.0 mg/kg. Therefore, it was concluded that a dose of 1.0 mg/kg should be recommended for a subsequent phase II trial of this novel agent [44].

A subsequent phase II study of mogamulizumab (0761–002 Study: ClinicalTrials.gov Identifier NCT 00920790) was conducted in patients with CCR4-positive relapsed ATL [45•]. The primary endpoint was the best ORR, and the secondary endpoints included the best response of each disease site such as peripheral blood ATL cells, skin and nodal/extranodal lesions as well as PFS and OS. It was planned for 25 patients to be enrolled for efficacy analysis, assuming the expected ORR of 30 % with a 5 % threshold response rate. Mogamulizumab was intravenously administered once a week for 8 weeks at a dose of 1.0 mg/kg. In this study, 28 patients in total were enrolled. Of these, 27 patients who received mogamulizumab were included in the safety analysis, and 26 patients, excluding 1 patient who was judged ineligible for enrollment after starting mogamulizumab administration, were evaluated in the efficacy analysis. Of the 27 patients who received mogamulizumab, 14 had acute type, 6 lymphoma type, and 7 chronic type with unfavorable prognostic factors. The best ORR was 50 % (13/26) including 8 CR. With the lower limit of the 95 % confidence interval (30 % to 70 %) exceeding the threshold response rate of 5 %, the clinical efficacy of mogamulizumab was confirmed. Responses according to disease sites were 100 % (of 13 patients, all CR) for peripheral blood, 63 % (of 8 patients, 3 CR and 2 PR) for skin, and 25 % (of 12 patients, 3 CR/CRu) for nodal and extranodal lesions. Median PFS and OS were 5.2 and 13.7 months, respectively. The best ORR was also calculated for each disease subtype, giving 43 % in patients with acute type (of 14 patients, 5 CR and 1 PR), 33 % in patients with lymphoma type (of 6 patients, 1 CR and 1 PR), and 83 % in patients with unfavorable chronic type (of 6 patients, 2 CR and 3 PR). Thus, it was demonstrated that mogamulizumab induced favorable responses in patients with any disease subtype of ATL. In addition, for each age group, the best ORR was 39 % (of 13 patients, 3 CR and 2 PR) in patients younger than 65 years, and 62 % (of 13 patients, 5 CR and 3 PR) in patients 65 years or older. The most common adverse events observed during the study were lymphopenia (96 %), neutropenia (52 %), and thrombocytopenia (52 %) as hematologic toxicity, and acute infusion reaction (89 %) and skin eruption (63 %) as non-hematologic toxicity. There was no death related to mogamulizumab in either the phase I or phase II study. Of 8 serious adverse events with a relationship to mogamulizumab in the phase I and II studies, 5 events including 4 cases of skin eruption and 1 case of Stevens-Johnson syndrome occurred during the phase II study. However, these reactions were manageable with supportive measures including corticosteroid or other drugs in all patients. Considering the seriousness of the disease, even skin eruption might be considered acceptable for the treatment of ATL by treating physicians, while close and appropriate follow-up of the event is necessary. Elucidation of the mechanism of skin eruption and preventive measures against it are awaited.

In the U.S., a phase I/II study (ClinicalTrials.gov Identifier: NCT00888927) in patients with relapsed or refractory PTCL including CTCL has been conducted [46]. Mogamulizumab was well tolerated at doses of 0.1–1.0 mg/kg in 42 patients including 1 with PTCL-NOS. MTD was not reached and thus 1.0 mg/kg was chosen for subsequent studies. A promising ORR of 42 % (of 38 evaluable patients with CTCL, 3 CR and 13 PR) was achieved, although expression of CCR4 on lymphoma cells was not mandatory for patient enrolment in this particular phase I/II study. Regarding subtypes of CTCL, ORR in Sezary syndrome (SS) patients was 50 % and in MF patients was 36 %. Eighty-seven percent of SS patients had a response in peripheral blood, with 50 % CR. Further study of mogamulizumab is warranted in patients with nodal PTCL as well as CTCL.

## Conclusions

It is evident that there are limitations to improvement of the treatment outcome of PTCL, especially ATL, with the currently available chemotherapeutic agents alone. Mogamulizumab has a less severe toxicity profile and induces a high response rate in patients with ATL, even in elderly patients. Therefore, it may provide an effective treatment option for the disease, especially for elderly patients who are not eligible for intensive chemotherapy or hematopoietic stem-cell transplantation.

**Fig. 1:**
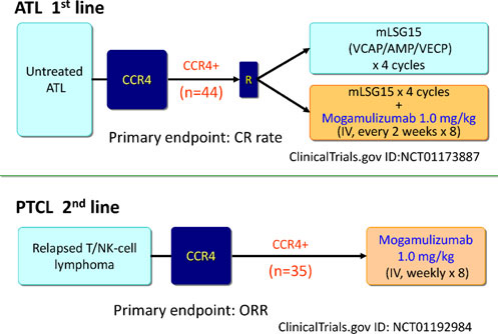
Ongoing clinical studies of mogamulizumab/KW-0761 for peripheral T-cell lymphomas in Japan

In Japan, based on the results of the clinical studies mentioned above, a single-arm phase II study of mogamulizumab monotherapy in patients with CTCL and PTCL (ClinicalTrials.gov Identifier: NCT01192984) and a randomized phase II study of dose-intensified combination chemotherapy (mLSG15 regimen) with or without mogamulizumab in untreated patients with ATL (ClinicalTrials.gov Identifier: NCT01173887) are being conducted as shown in Fig. 1. Patient enrollment has already been completed in these phase II studies. In the U.S., based on the aforementioned phase I/II study in patients with relapsed or refractory CTCL, a pivotal phase III study in patients with relapsed or refractory CTCL is being planned. In conclusion, mogamulizumab is expected to provide new, promising treatment options in patients with ATL and other T-cell lymphomas.
